# Systematic review and network meta-analyses of third-line treatments for metastatic colorectal cancer

**DOI:** 10.1007/s00432-020-03315-6

**Published:** 2020-07-27

**Authors:** Thomas Walter, Neil S. Hawkins, Richard F. Pollock, Fabien Colaone, Suki Shergill, Paul J. Ross

**Affiliations:** 1 Cancer Research Center of Lyon, University of Lyon, Claude Bernard University, Lyon, France; 2 Service d’oncologie médicale, Hôpital E. Herriot, Hospices Civils de Lyon, Lyon, France; 3 Institute of Health and Wellbeing, University of Glasgow, Glasgow, UK; 4 Covalence Research Ltd, 51 Hayes Grove, London, SE22 8DF UK; 5 Sirtex Medical United Kingdom Ltd, London, UK; 6 Department of Medical Oncology, Guy’s and St Thomas’ NHS Foundation Trust, London, UK; 7 Department of Oncology, King’s College Hospital NHS Foundation Trust, London, UK

**Keywords:** Colorectal cancer, Neoplasm metastasis, Meta-analysis

## Abstract

**Background:**

Limited treatment options are available in chemotherapy-refractory metastatic colorectal cancer (mCRC). The objective was to conduct a systematic literature review (SLR) and exploratory network meta-analysis (NMA) to compare the tolerability and effectiveness of SIRT with Y-90 resin microspheres, regorafenib, TAS-102 (trifluridine/tipiracil), and best supportive care (BSC) as third-line treatment in patients with mCRC.

**Methods:**

An SLR was conducted to identify studies comparing two or more of the treatments and reporting overall survival (OS), progression-free survival, tumor response, or adverse event (AE) incidence. An exploratory NMA was conducted to compare hazard ratios (HRs) for OS using Markov chain Monte Carlo (MCMC) techniques.

**Results:**

Seven studies were identified in the SLR: two double-blind randomized-controlled trials (RCT) for each drug, one open-label RCT, and two non-randomized comparative studies for SIRT. Patient selection criteria differed between studies, with SIRT studies including patients with liver-dominant colorectal metastases. Nausea and vomiting were more frequent with TAS-102 than regorafenib or SIRT; diarrhea was more common with TAS-102 and regorafenib than SIRT. The exploratory NMA suggested that all active treatments improved OS, with HRs of 0.48 (95% CrI 0.30–0.78) for SIRT with Y-90 resin microspheres, 0.63 (0.38–1.03) for TAS-102, and 0.67 (0.40–1.08) for regorafenib each compared to BSC.

**Conclusions:**

Regorafenib, TAS-102 and SIRT using Y-90 resin microspheres are more effective than BSC in third-line treatment of mCRC; however, study heterogeneity made comparisons between active treatments challenging. SIRT is a viable treatment for third-line mCRC and its favorable AE profile should be considered in the therapeutic decision-making process.

**Electronic supplementary material:**

The online version of this article (10.1007/s00432-020-03315-6) contains supplementary material, which is available to authorized users.

## Background

Colorectal cancer (CRC) is the third most common cancer in the world by incidence, but second in terms of mortality, with an estimated 881,000 deaths in 2018 (Bray et al. 2018). Furthermore, the annual incidence is estimated to rise to over 3 million cases by 2040 (Ferlay et al. 2019). Metastases are reported in at least half of all CRC cases, and population-based studies report that liver metastases develop in approximately 60–70% of all cases of metastatic CRC (mCRC), with metastases restricted to the liver in approximately 35–55% of patients with mCRC (van der Geest et al. 2015; Adam et al. 2012; van der Pool et al. 2012; Mekenkamp et al. 2010; Kumar et al. 2014; Sadahiro et al. 2013; Kumar et al. 2013; Oh et al. 2015; Kennecke et al. 2014). Many patients with mCRC eventually become insensitive or unresponsive to chemotherapy (chemorefractory) or cannot tolerate multiple cycles of chemotherapy (chemotherapy-intolerant). In clinical practice, of all mCRC patients receiving first-line chemotherapy, approximately 50% go on to receive second-line chemotherapy, and of these patients, approximately 25% go on to receive third-line chemotherapy (Abrams et al. 2014; Zafar et al. 2009).

Limited treatment options are available in chemorefractory mCRC. Regorafenib (STIVARGA^®^; Bayer, Leverkusen, Germany) received US Food and Drug Administration (FDA) approval in 2012, and EMA approval in 2013; trifluridine–tipiracil (TAS-102, LONSURF^®^; Servier, Suresnes, France, and Taiho, Princeton, NJ) received FDA approval in 2015 and EMA approval in 2016. Both treatments are approved in Europe for patients with mCRC “who have been previously treated with, or are not considered candidates for, available therapies” including fluoropyrimidine-, oxaliplatin- and irinotecan-based chemotherapies, anti-VEGF agents, and anti-epidermal growth factor receptor (EGFR) agents (Bayer 2018; Les Laboratoires Servier 2016). Standard chemotherapy regimens for these patients typically consist of two lines of therapy combining the above agents; therefore, regorafenib and TAS-102 are considered as third-line therapy. While anti-EGFR re-challenge strategies are emerging as a treatment option for patients with RAS wild-type mCRC, options for patients with RAS-mutated mCRC remain limited and additional third-line therapies are a strong medical need (Van Cutsem et al. 2015; Mauri et al. 2019).

For patients failing third-line therapy, or not eligible for current third-line treatments, no other systemic therapy options are currently available and disease management is restricted to best supportive care (BSC). In cases of disease progression after two lines of treatment in mCRC, BSC is associated with median survival times of 4–6 months (Foubert et al. 2014).

SIRT with SIR-Spheres Y-90 resin microspheres is a treatment option for patients with liver-only or liver-dominant mCRC, and is recommended by European Society for Medical Oncology (ESMO) 2016 Guidelines for patients who are refractory or intolerant to chemotherapy (category B recommendation for yttrium-90 resin microspheres) and National Comprehensive Cancer Network (NCCN) Guidelines v3.2020 (category 2A recommendation for “arterially directed catheter therapy, and in particular yttrium-90 microsphere selective internal radiation”) in this indication (Van Cutsem et al. 2016; Benson et al. 2020). The current guidelines do not, however, offer recommendations on the respective positions of SIRT with SIR-Spheres Y-90 resin microspheres, regorafenib, or TAS-102 in the therapeutic strategy, especially for patients with colorectal liver metastases, as the guideline committees did not identify evidence comparing these interventions.

The objective of the present study was to conduct a systematic literature review with a descriptive analysis of the adverse event and overall survival (OS) data, and to conduct an exploratory network meta-analysis (NMA) comparing the relative clinical effectiveness and tolerability of SIRT with SIR-Spheres Y-90 resin microspheres, regorafenib, TAS-102, and BSC as third-line treatment in patients with mCRC.

## Methods

The study was conducted in three stages: a systematic literature review designed to identify studies comparing two or more of the following interventions: SIR-Spheres Y-90 resin microspheres, TAS-102, regorafenib, or BSC; a descriptive analysis of the reported adverse event rates and median OS; and an exploratory network meta-analysis (NMA) comparing hazard ratios (HR) for OS.

### Systematic review

A priori inclusion and exclusion criteria were developed using the Population, Intervention, Comparator, Outcome, Study Design (PICOS) methodology (Table 1). The PICOS criteria were designed to identify any studies investigating the use of BSC, TAS-102, regorafenib, or SIRT with Y-90 resin microspheres in third-line treatment of mCRC reporting OS, progression-free survival, tumor response, or adverse events. The study design criterion was structured to include comparative non-randomized studies of SIRT in addition to randomized-controlled trials on the basis of opinion from clinical experts solicited during the design of the literature search strategy, noting that there was likely to be a paucity of RCT data on SIRT in third-line mCRC.

**Table 1:** Population, intervention, comparators, outcomes, and study design (PICOS) criteria employed in the systematic literature review

Population	Randomized and non-randomized interventional and comparative observational studies of patients with chemotherapy-refractory or intolerant (third-line) mCRC including treatment with SIR-Spheres Y-90 resin microspheres, TAS-102 (trifluridine/tipiracil), and regorafenib
Intervention	SIR-Spheres Y-90 resin microspheres
Comparators	TAS-102 (trifluridine/tipiracil) Regorafenib Best supportive care
Outcomes	Overall survival Progression-free survival Response Specific adverse events as reported
Study design	Comparative studies of SIRT with Y-90 resin microspheres, TAS-102, regorafenib, and best supportive care

The PubMed, EMBASE, and Cochrane Collaboration databases, and clinicaltrials.gov were searched using standard filters to identify clinical trials (Supplementary Tables 1 and 2). After removal of duplicate studies, titles and abstracts of the unique articles were screened by two independent reviewers and any conflicts were resolved by a third reviewer. Screening of the full-text copies of studies included after title and abstract screening was then conducted, and data were extracted from the papers included after full-text screening. Data on adverse events were extracted in two aggregated categories: Common Terminology Criteria for Adverse Events (CTCAE) Grades 1 and 2, and CTCAE Grades 3+. Adverse event data were only presented for those events occurring in > 5% of patients with Grade 1 and 2 events or > 2% of patients with Grade 3+ events in two or more studies, and those events that were potentially related to SIRT: bile duct complications, gastritis, gastrointestinal (GI) ulcers, radiation pneumonitis, and radioembolization-induced liver disease (REILD). Full-text screening and data extraction results were validated by an independent reviewer.

### Evidence synthesis

Adverse event rates and median survival were summarized, and an exploratory NMA was conducted to provide the estimates of comparative effectiveness for the specified outcomes. The network meta-analysis was conducted using published methodology (Hawkins et al. 2016). The model parameters were estimated using Markov chain Monte Carlo (MCMC) techniques as implemented in JAGS 4.3.0 (Plummer 2003). Three chains were run starting from different initial values. Models were initially run for 20,000 iterations as a burn-in period and a further 20,000 iterations for estimation and convergence. Further iterations were considered if the Monte Carlo sampling error and convergence were judged not to be adequate. Convergence was assessed using Brooks–Gelman–Rubin plots and by examining history plots (the informal ‘blue finger test’) (Brooks and Gelman 1998). Adequacy of Monte Carlo sampling error was judged using the *R*_hat_ statistic and model fit was compared using the Deviance Information Criterion (DIC) (Brooks and Gelman 1998).

The exploratory NMA was conducted using HRs for OS assuming that placebo, BSC, and 5-FU monotherapy could be treated as a common BSC comparator. Both fixed and random effects pairwise meta-analyses were also undertaken, with the random effects meta-analyses incorporating an informative prior based on a review of previous meta-analyses (Turner et al. 2012). The effect of including data from non-randomized studies of SIRT was investigated through a series of analyses in which the variance around the mean HRs from the non-randomized studies was inflated (and hence the precision decreased) using a factor 1/*w*_*j*_ where 0 < *w*_*j*_ < 1 (Efthimiou et al. 2017). In the primary analysis, *w*_*j*_ was set to 1, assigning equal weight to randomized and non-randomized studies. A validation analysis was then conducted with *w*_*j*_ ≈ 0, approximating an analysis of RCTs only, with sensitivity analyses conducted with *w*_*j*_ = 0.2, 0. 25, $0.3\dot{3}$, 0.5, and $0.6\dot{6}$.

## Results

### Search results

The literature searches retrieved 1524 studies, of which 1334 were unique (Supplementary Fig. 1). Title and abstract screening resulted in the exclusion of 1294 studies, leaving 40 studies for full-text screening. After screening of the full-text articles, seven studies were ultimately deemed eligible to be included in the analyses. The studies consisted of: one randomized open-label trial and two non-randomized studies comparing SIRT with BSC (Hendlisz et al. 2010; Bester et al. 2012; Seidensticker et al. 2012), two randomized double-blind trials comparing TAS-102 with placebo (Yoshino et al. 2012; Mayer et al. 2015), and two randomized double-blind trials comparing regorafenib with placebo (the RECOURSE and CONCUR) (Grothey et al. 2013; Li et al. 2015). While one of the non-randomized studies of SIRT (Bester et al. 2012) included patients with both liver metastases secondary to colorectal cancer (66% of subjects) and secondary to other sites (34%), the study was ultimately included as the results from the colorectal primary subgroup were reported separately from the overall population (Bester et al. 2012).

### Study characteristics

The key characteristics and designs of the included studies are summarized in Table 2. The SIRT studies included an open-label randomized trial and two non-randomized comparative studies. The TAS-102 and regorafenib studies were double-blind randomized trials. Differences in the nature of SIRT and BSC interventions received by patients prevented blinding.

**Table 2:** Study designs

Author	Study type	Intervention	Comparator	Location(s)
Seidensticker 2012	Non-randomized observational study [matched analysis based on prior treatment history, tumor burden, liver involvement., synchronous versus metachronous metastases, alkaline phosphatase (ALP) increase, and carcinoembryonic antigen (CEA)]	SIRT (*n* = 29)	BSC (*n* = 29)	Germany
Hendlisz 2010 (NCT00199173)	Randomized open-label trial	SIRT + 5-fluorouracil (*n* = 21)	5-fluorouracil with cross-over to SIRT upon progression at investigators' discretion (*n* = 23)	Belgium
Bester 2012	Non-randomized interventional study of SIRT with control group formed of patients ineligible for SIRT patients (multivariable regression including potential prognostic factors conducted for survival analysis only). CRC patients only	SIRT (*n* = 224)	BSC (*n* = 29)	Australia
Yoshino 2012 (JapicCTI-090880)	Randomized double-blind trial	TAS-102 at 35 mg/m^2^ twice daily (*n* = 112)	Placebo + BSC (*n* = 57). Cross-over not permitted	Japan
Mayer 2015 (RECOURSE, NCT01607957)	Randomized double-blind trial	TAS-102 at 35 mg/m^2^ twice daily (*n* = 534)	Placebo + BSC (*n* = 266). Cross-over not permitted	Japan, United States, Europe, and Australia
Li 2015 (CONCUR, NCT01584830)	Randomized double-blind trial	Regorafenib at 160 mg/day (*n* = 136)	Placebo + BSC (*n* = 68)	China, Hong Kong, South Korea, Taiwan, and Vietnam
Grothey 2013 (CORRECT, NCT01103323)	Randomized double-blind trial	Regorafenib at 160 mg/day (*n* = 505)	Placebo + BSC (*n* = 255). Crossover not permitted	North America, western Europe, Israel, Australia, Asia, and Eastern Europe

*BSC* best supportive care, *CRC* colorectal cancer, *SIRT* selective internal radiation therapy

The TAS-102 and regorafenib studies were placebo-controlled studies in which patients also received BSC. Bester 2012 and Seidensticker 2012 had control arms in which patients received BSC. Although Hendlisz 2010 was a randomized trial, it compared SIRT with concurrent 5-fluorouracil (5-FU) versus 5-FU alone, allowing cross-over from the 5-FU alone arm to SIRT on progression at the investigators’ discretion. Since patients enrolled in Hendlisz 2010 had already progressed on a prior treatment regimen including 5-FU and 5-FU is not typically prescribed as monotherapy for mCRC, the control arm was assumed to be similar to BSC, although the impact of 5-FU itself is uncertain. Notably, 70% of patients in the 5-FU arm did cross over, including ten patients who received SIRT, potentially confounding overall survival outcomes of the trial (Hendlisz et al. 2010).

The demographics and clinical characteristics of study subjects are summarized in Table 3. The study subjects had a similar mean age across studies. In general, there was a slight excess of males included in the studies. Performance status was generally preserved with most patients (95–100%) having Eastern Cooperative Oncology Group (ECOG) scores of 0 or 1.

**Table 3:** Summary of subject demographics

	*N*	Median age (years)	Male (%)	ECOG	ECOG	ECOG	KRAS mutation (%)	EHD (%)	Multiple metastatic organs (%)
				PS 0 (%)	PS 1 (%)	PS 2 (%)			
SIRT versus BSC									
Bester 2012 SIRT	224	67*	63	85	NR	NR	NR	38	NR
Bester 2012 BSC	51**	66*	69	NR	NR	NR	NR	33	NR
Hendlisz 2010 SIRT	21	62	48	71	24	4.8	NR	0^†^	0^†^
Hendlisz 2010 BSC	23	62	78	74	22	4.3	NR	0^†^	0^†^
Seidensticker 2012 SIRT	29	62*	76	NR	NR	80^#^	NR	48.3	NR
Seidensticker 2012 BSC	29	61*	79	NR	NR	80^#^	NR	48.3	NR
TAS-102 versus BSC									
Mayer 2015 TAS-102	534	63	61	56	44	NR	51	NR	NR
Mayer 2015 BSC	266	63	62	55	45	NR	51	NR	NR
Yoshino 2012 TAS-102	112	63	57	64	33	2.7	40	NR	78
Yoshino 2012 BSC	57	62	49	61	37	1.8	46	NR	81
Regorafenib versus BSC									
Grothey 2013 regorafenib	505	61	62	52	48	NR	54	NR	NR
Grothey 2013 BSC	255	61	60	57	43	NR	62	NR	NR
Li 2015 regorafenib	136	58	63	26	74	NR	34	NR	79
Li 2015 BSC	68	56	49	22	78	NR	26	NR	78

*BSC* best supportive care, *ECOG PS* Eastern Cooperative Oncology Group performance status, *EHD* extrahepatic disease aside from the primary site, *KRAS* Kirsten rat sarcoma, *NR* not reported, *SIRT* selective internal radiation therapy

*Mean age, **Also includes patients with other primary tumors

^†^Extrahepatic disease listed as exclusion criterion

^#^Karnofsky index, not ECOG PS

The proportions of patients’ exposure to previous chemotherapy is summarized in Table 4. Almost all patients had received previous systemic therapy and all patients included in the selected studies were deemed refractory or intolerant to other available systemic agents available at the time of enrolment. Prior bevacizumab use varied between the studies; all patients in the Mayer et al. and Grothey et al. studies had received previous treatment with bevacizumab, with approximately 50% and 80% of patients having received bevacizumab in the Seidensticker et al. and Yoshino et al. studies, respectively. Reporting of the number of treatment lines received by the enrolled patients was inconsistent across studies, making quantitative comparisons between the different study populations difficult. The proportion of patients with extrahepatic disease (aside from the primary tumor) was not consistently reported across studies, but Bester et al. reported extrahepatic disease in 38% of patients receiving SIRT and 33% of patients receiving BSC; Yoshino et al. reported a total of 78% of patients receiving TAS-102 with two or more metastatic organs, compared with 81% of patients receiving placebo; and Hendlisz et al. specified extrahepatic disease as an exclusion criterion (Hendlisz et al. 2010; Bester et al. 2012; Seidensticker et al. 2012; Yoshino et al. 2012).

**Table 4:** Summary of prior systemic therapies

	Prior chemotherapy regimens (%)										Irinotecan (%)	Oxaliplatin (%)	Bevacizumab (%)	Cetuximab (%)	Regorafenib (%)
	Any	1	1–2	2	3	≥ 3	4	≥ 4	5	6					
SIRT versus BSC															
Bester 2012 SIRT	91	NR	NR	NR	NR	NR	NR	NR	NR	NR	NR	NR	NR	NR	NR
Bester 2012 BSC	92	NR	NR	NR	NR	NR	NR	NR	NR	NR	NR	NR	NR	NR	NR
Hendlisz 2010 SIRT	NR	NR	NR	NR	NR	NR	NR	NR	NR	NR	62	19	NR	NR	NR
Hendlisz 2010 BSC	NR	NR	NR	NR	NR	NR	NR	NR	NR	NR	87	8.7	NR	NR	NR
Seidensticker 2012 SIRT	NR	0	NR	28	31	NR	34	NR	3.4	3.4	90	90	52	52	NR
Seidensticker 2012 BSC	NR	0	NR	24	38	NR	24	NR	10	3.4	100	90	48	66	NR
TAS-102 versus BSC															
Mayer 2015 TAS-102	NR	NR	NR	18	22	NR	NR	60	NR	NR	100	100	100	NR	17
Mayer 2015 BSC	NR	NR	NR	17	20	NR	NR	63	NR	NR	100	100	100	NR	20
Yoshino 2012 TAS-102	NR	NR	NR	15	NR	85	NR	NR	NR	NR	100	100	78	63	NR
Yoshino 2012 BSC	NR	NR	NR	23	NR	77	NR	NR	NR	NR	100	100	82	63	NR
Regorafenib versus BSC															
Grothey 2013 regorafenib	NR	NR	27	NR	25	NR	NR	49	NR	NR	NR	NR	100	NR	NR
Grothey 2013 BSC	NR	NR	25	NR	28	NR	NR	47	NR	NR	NR	NR	100	NR	NR
Li 2015 regorafenib	NR	NR	NR	23	24	NR	NR	54	NR	NR	NR	NR	NR	NR	NR
Li 2015 BSC	NR	NR	NR	21	28	NR	NR	51	NR	NR	NR	NR	NR	NR	NR

*BSC* best supportive care, *NR* not reported, *SIRT* selective internal radiation therapy

Due to the limited data available on response and progression in the SIRT studies, only results for adverse event incidence and OS are presented.

### Adverse events

**Fig. 1:**
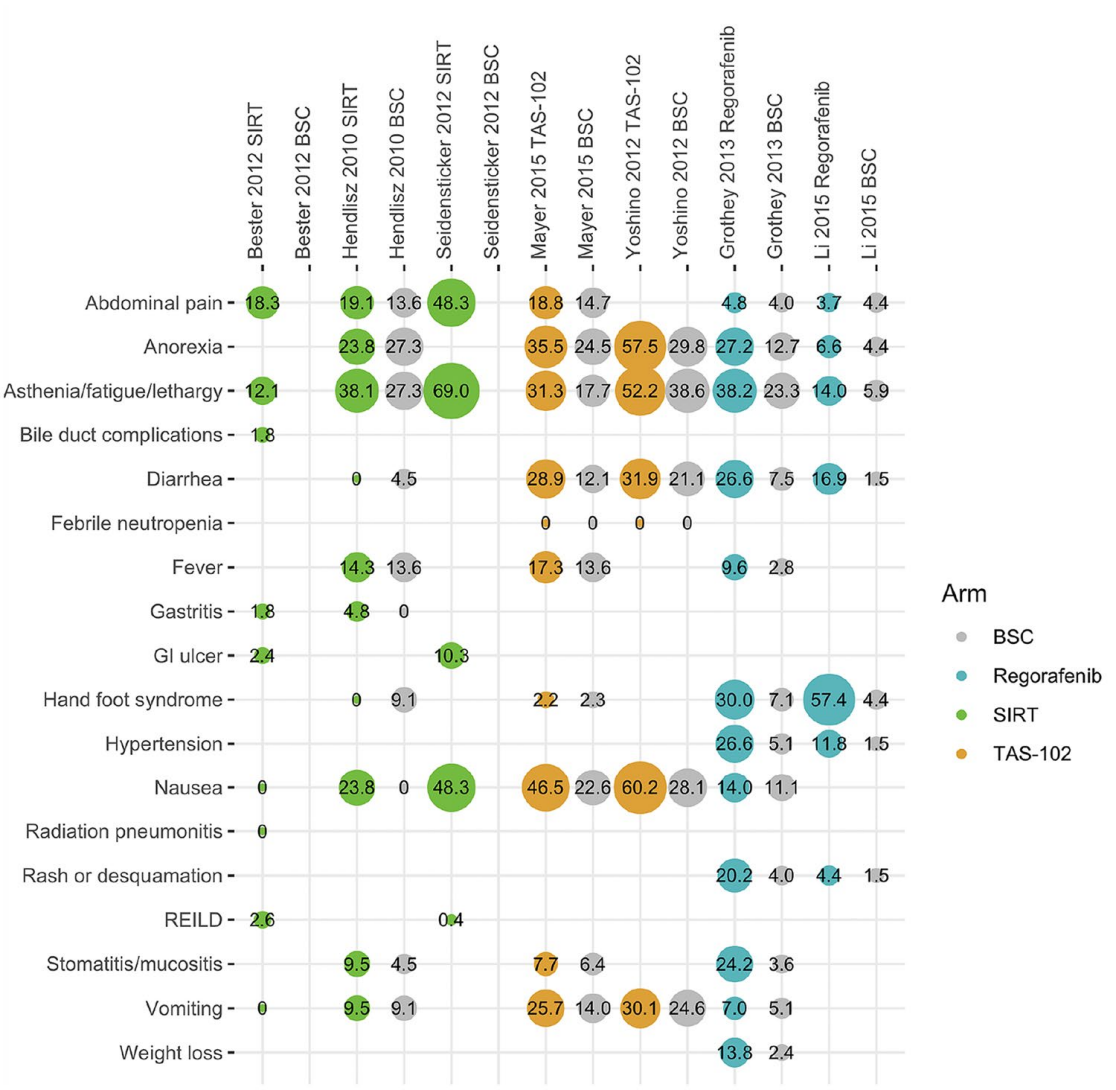
Summary of grade 1 or 2 adverse events occurring in > 5% of patients in two or more studies. *BSC* best supportive care, *GI* gastrointestinal, *REILD* radioembolization-induced liver disease, *SIRT* selective internal radiation therapy. Note: areas of circles represent the proportion of study subjects reporting specific symptoms

**Fig. 2:**
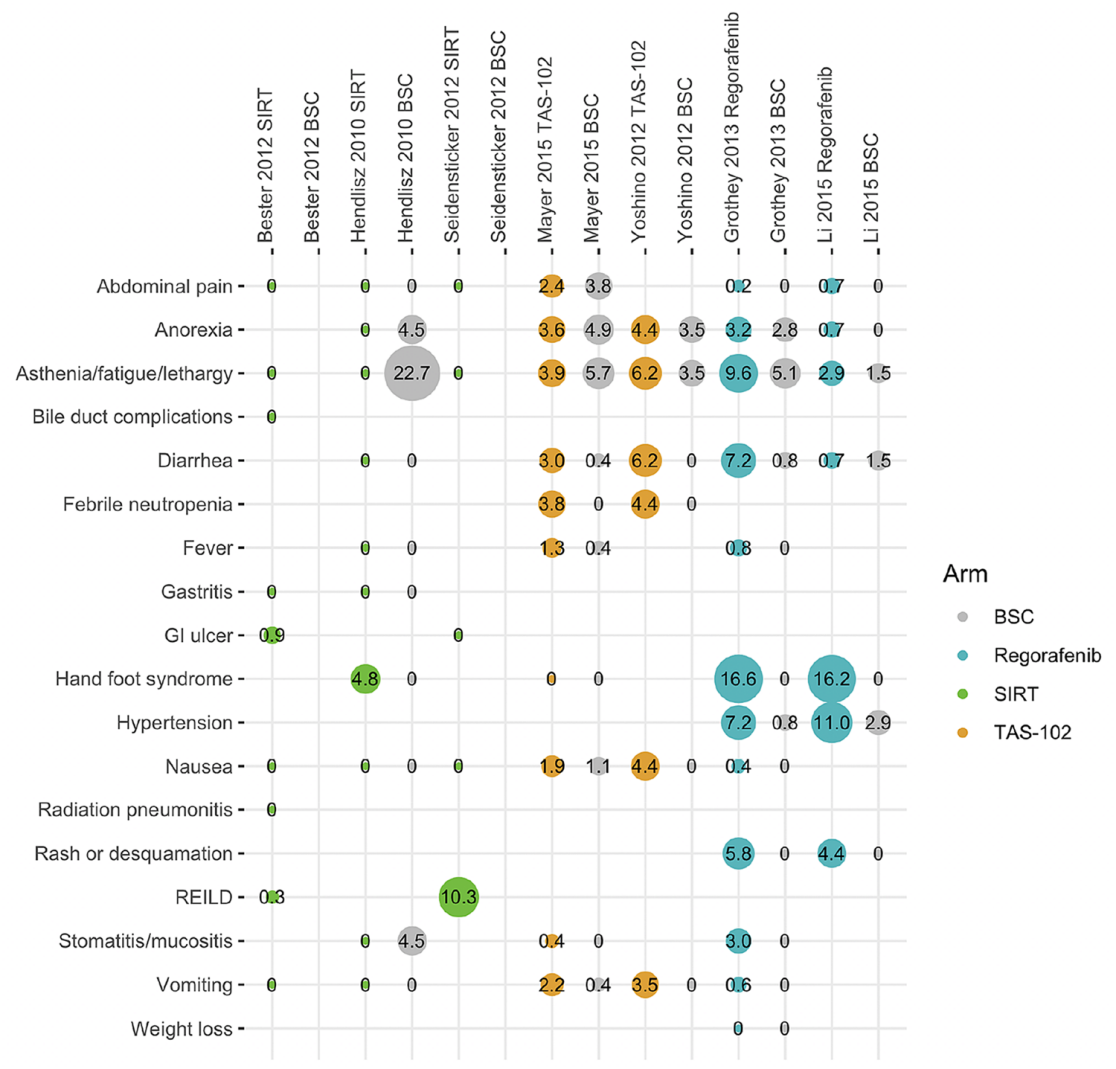
Summary of grade 3, 4, and 5 adverse events occurring in > 2% of patients in two or more studies

The percentages of patients reporting symptoms or non-laboratory signs of a grade 1 or 2 adverse event (with a prevalence > 5% in at least two studies) is shown in Fig. 1, with the corresponding percentages for grade 3, 4, or 5 events (with a prevalence > 2% in at least two studies) presented in Fig. 2. There was some evidence of increased rates of diarrhea with TAS-102 and regorafenib compared with SIRT and increased rates of nausea and vomiting with TAS-102 compared with regorafenib and SIRT. Regorafenib was also associated with increased rates of hand–foot syndrome, hypertension, and rash or desquamation. Radioembolization-induced liver disease (REILD) was only reported in the Bester et al. and Seidensticker et al. studies in which the incidence rates at grade 3+ were 0.3% and 10.3%, respectively. The only other potentially SIRT-related adverse event (as defined in the present study) that occurred in > 5% of patients was grade 1–2 GI ulcer, which occurred in 10.3% of patients in the Seidensticker et al. study. Reporting of laboratory test abnormalities was highly inconsistent throughout the evidence base and events were not, therefore, aggregated or analyzed for comparison.

### Overall survival

**Fig. 3:**
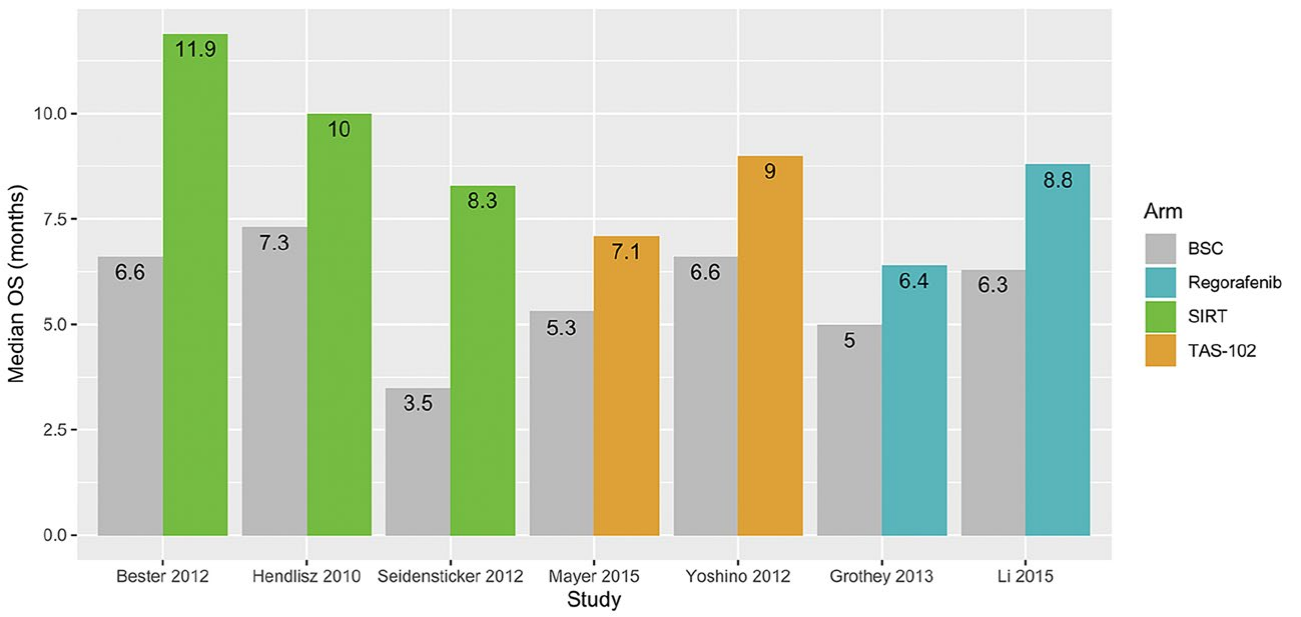
Median overall survival (months) in each study arm. *BSC* best supportive care, *OS* overall survival, *SIRT* selective internal radiation therapy

The reported median survival for each study arm is shown in Fig. 3. In all studies, median survival is less than 12 months and SIRT, TAS-102, or regorafenib is each associated with prolonged survival (median OS: 6.4–11.9 months) compared to non-active, BSC (median OS: 3.5–7.3 months). The longest survival on BSC was observed in the Hendlisz 2010 trial of SIRT, in which patients in the control group were allowed to cross-over to active treatment after progression for ethical reasons (Hendlisz et al. 2010).

**Fig. 4:**
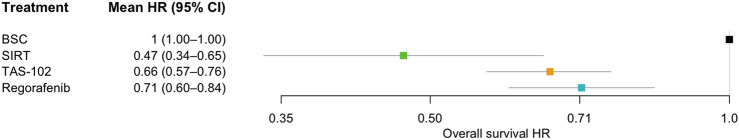
Overall results from the fixed effects network meta-analysis showing the mortality hazard ratio for each treatment relative to a reference of 1 with best supportive care. *BSC* best supportive care, *HR* hazard ratio, *SIRT* selective internal radiation therapy

**Fig. 5:**
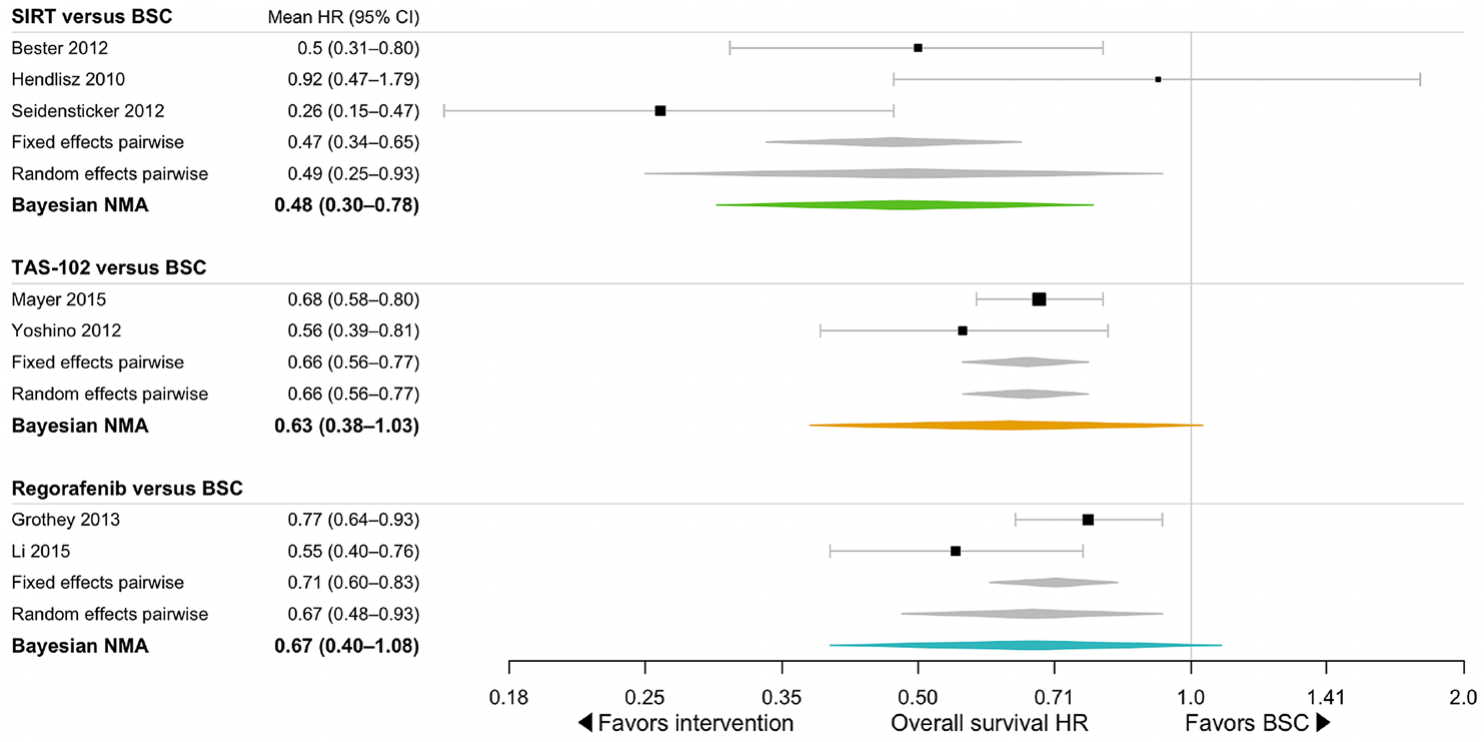
Individual study results for the random effects network meta-analysis. *BSC* best supportive care, *HR* hazard ratio, *OS* overall survival, *SIRT* selective internal radiation therapy

**Fig. 6:**
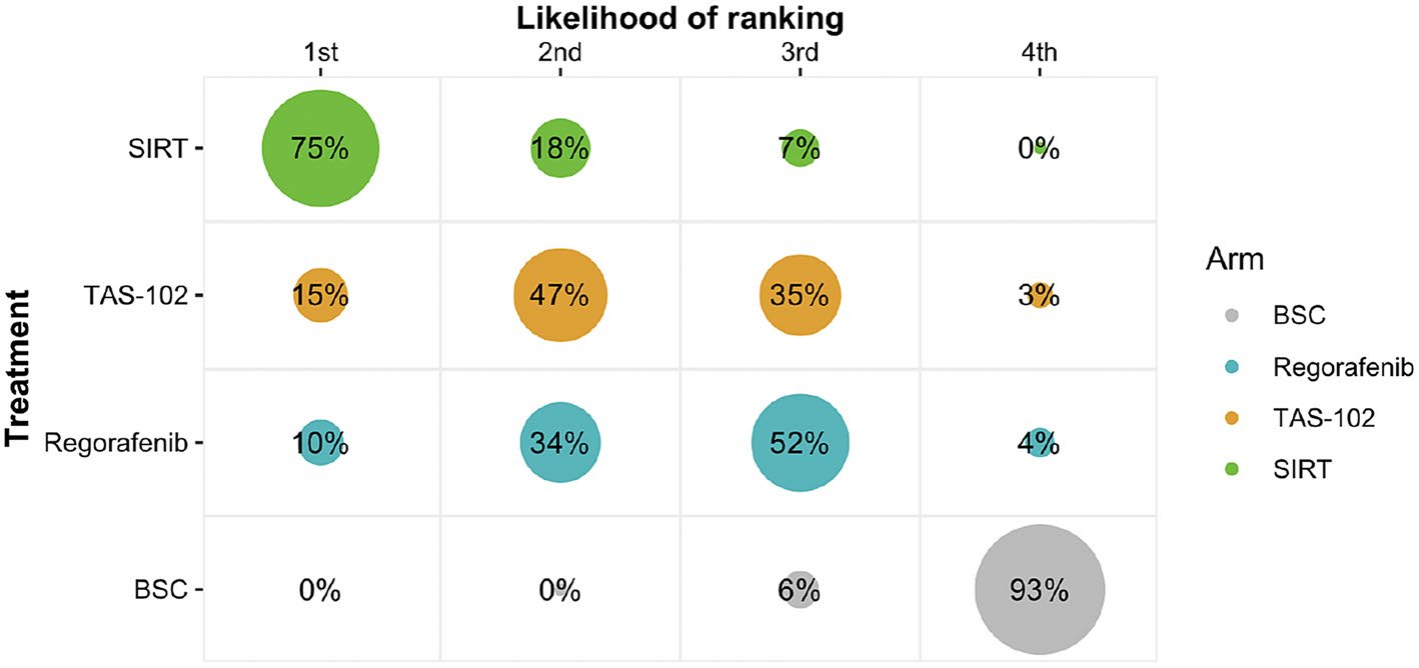
Probability of treatment rankings based on the random effects network meta-analysis. *BSC* best supportive care, *SIRT* selective internal radiation therapy

Brooks–Gelman–Rubin, trace, and marginal density plots showed that the NMA converged on a solution within the 20,000 iterations after the burn-in period (Supplementary Fig. 2 and 3). The results of the fixed effects NMA are shown in Fig. 4 and the results of the fixed and random effects pairwise meta-analyses, and the random effects NMA are shown in Fig. 5. In the pairwise fixed effects meta-analyses, all active treatments were shown to improve OS, with mortality hazard ratios of 0.47 (95% CrI 0.34–0.65) for SIRT with Y-90 resin microspheres, 0.66 (0.56–0.77) for TAS-102, and 0.71 (0.60–0.83) for regorafenib each compared to BSC. Hazard ratios were similar in the pairwise random effects meta-analyses, at 0.49 (95% CrI 0.25–0.93) for SIRT, 0.66 (0.56–0.77) for TAS-102, and 0.67 (0.48–0.93) for regorafenib, each compared to BSC. Rank probabilities from the random effects NMA showed that there was a 75% probability of SIRT with Y-90 resin microspheres being the most effective treatment, compared with 15% for TAS-102, and 10% for regorafenib (Fig. 6); there was a 0% probability that BSC would be the optimal treatment in terms of OS.

Sensitivity analyses in which the variance around the OS hazard ratios from the two non-randomized studies of SIRT was inflated showed that the 95% CrI for SIRT relative to BSC only crossed 1 when the variance was four times greater than in the base case analysis (*w*_*j*_ = 0.25; Supplementary Fig. 4). With a fourfold increase in variance around the hazard ratios from non-randomized studies, the mean hazard ratio for OS with SIRT relative to BSC was 0.59 (95% CrI 0.32–1.06).

While SIRT was ranked first based on OS, with TAS-102 second and regorafenib third (Fig. 6), there was substantial uncertainty as to the ranking of treatments due to the heterogeneity in study designs and the variation in comparator treatments. Although results of the network meta-analysis must be considered with caution, they demonstrate that all three active treatments are associated with a statistically significant reduction in the mortality risk compared to BSC.

## Discussion

The present study is the first meta-analysis of SIRT, regorafenib, and TAS-102 for patients with chemotherapy-refractory or -intolerant mCRC. Previous meta-analyses have observed similar results for TAS-102 and regorafenib, at a starting dose of 160 mg/day, with no statistically significant difference in OS between the two drug regimens (Abrahao et al. 2018). Although both drugs are associated with increased incidence of treatment-emergent grade 3–4 AEs, the types of AEs associated with each drug are different. Regorafenib is frequently associated with AEs commonly reported for tyrosine kinase inhibitors, such as diarrhea, hand–foot skin reaction, or hypertension, while TAS-102 is more frequently associated with hematological abnormalities, as also evidenced in the present review. Regorafenib was reportedly associated with higher toxicity than TAS-102 overall (Abrahao et al. 2018).

The recently published ReDOS trial of regorafenib, comparing standard administration at a starting dose of 160 mg/day versus dose escalation at a starting dose of 80 mg/day, reported increased OS for the dose escalation strategy with lower incidence of grade 3 adverse events commonly associated with regorafenib (Bekaii-Saab et al. 2019). Based on published meta-analyses and outcomes of the present NMA, it is uncertain whether this OS increase would match the magnitude of clinical benefit observed for SIRT with Y-90 resin microspheres (Sonbol et al. 2019).

A key limitation of the present review was that the included studies of SIRT with Y-90 resin microspheres recruited exclusively subjects with colorectal metastases to the liver (with or without other extrahepatic disease), consistent with the liver-directed nature of the intervention, while studies of systemic treatment with regorafenib or TAS-102 included patients with any metastatic location. This difference in study design warrants caution when comparing median OS outcomes across the studies. This exploratory NMA was undertaken comparing HRs for OS, which are expected to capture differences in baseline characteristics across studies, as patients in the control group of each study of SIRT also have liver-dominant or liver-only metastatic disease. Nevertheless, it is worth noting that the median OS for patients receiving BSC was comparable in all studies, regardless of these differences in selection criteria, and that the lowest median OS was observed in the BSC group of the Seidensticker et al. 2012 study which only included patients with liver-dominant metastases (Seidensticker et al. 2012). This may reflect the prognostic importance of the liver and of liver metastases for patients with chemotherapy-refractory or -intolerant mCRC (Hendlisz et al. 2010).

Although the potential for confounding due to sample selection bias in the observational studies of SIRT included in this review should be noted, OS outcomes were consistent with those reported in large single-arm observational studies of regorafenib, TAS-102 and SIRT using Y-90 resin microspheres (de Groot J 2018; Andersen et al. 2019; Golfieri et al. 2015; Cosimelli et al. 2010; Kennedy et al. 2015; Saxena et al. 2015; Tohme et al. 2014; Lahti et al. 2015; Fendler et al. 2013; Maleux et al. 2016; Sofocleous et al. 2015; Nace et al. 2011). In ten identified studies of SIRT using Y-90 resin microspheres published between 2010 and 2019 which enrolled 50 or more patients with chemotherapy-refractory or -intolerant, liver-dominant colorectal metastases, median OS was between 6.9 and 13.8 months (median 10.2 months, pooled mean 9.9 months, and *N* = 1476). Sensitivity analysis also showed the findings to be insensitive to substantial increases in the modeled variance around the results from the non-randomized studies, with a fivefold increase in variance still associated with an average OS hazard ratio of 0.62 with SIRT relative to BSC. Other, more sophisticated techniques could have been employed to investigate the effect of including non-randomized studies in the analysis such as third-level hierarchical Bayesian modeling or utilizing data from randomized studies to modify the priors; (Efthimiou et al. 2017; Cameron et al. 2015); however, given that the non-randomized study data were confined to the single network edge comparing SIRT versus BSC, these techniques would have been unlikely to provide further insights into any bias arising from the non-randomized study designs.

Regorafenib and TAS-102 are recommended in ESMO, NCCN, French Intergroup, and Spanish consensus clinical guidelines for patients failing fluoropyrimidine-, oxaliplatin-, and irinotecan-based first- and second-line chemotherapy regimens (Van Cutsem et al. 2016; Benson et al. 2020; Phelip et al. 2019; Vera et al. 2019). SIRT using Y-90 resin microspheres is recommended in the ESMO and French guidelines in a similar setting, for patients with liver-only or liver-dominant colorectal metastases who are refractory or intolerant to chemotherapy, while NCCN guidelines are less specific about the recommended position for SIRT in the therapeutic strategy. These guidelines and the OS outcomes of the present review support offering patients presenting with colorectal liver metastases refractory to first- and second-line chemotherapy treatment with either systemic therapy options or SIRT with Y-90 resin microspheres depending on clinician and patient preference. In considering the mCRC armamentarium, it is worth mentioning the options available to specific sub-groups of patients with mCRC. Patients with BRAF V600E mutant mCRC (approximately 10% of mCRC cases) may be suitable for BRAF targeted therapies, such as encorafenib in combination with cetuximab, which received FDA in this indication after prior therapy in April 2020 (US Food and Drug Administration 2020; Kopetz et al. 2019). Numerous other BRAF pathway inhibitors, including dabrafenib, vemurafenib, and trametinib, are currently undergoing trials either alone or in combination with EGFR and/or MEK inhibitors in patients with BRAF V600E mutant mCRC (Ducreux et al. 2019). Moreover, patients with mismatch repair deficient (dMMR) and/or microsatellite instability-high (MSI-H) (approximately 3–5% of mCRC cases) may benefit from immune checkpoint inhibitors such as pembrolizumab, or nivolumab either alone or in combination with ipilimumab, all of which were approved by the FDA in 2017–2018 for use in patients with dMMR/MSI-H mCRC having progressed following treatment with fluoropyrimidine, oxaliplatin, and irinotecan (Kamatham et al. 2019; Le et al. 2020; Overman et al. 2018). Enrollment of patients in trials of these and other agents may also be a viable option for patients with advanced mCRC refractory to multiple lines of chemotherapy, with the NCCN specifically noting in the CRC guidelines their belief that “the best management for any patient with cancer is in a clinical trial” (Benson et al. 2020).

With regard to the sequencing of locoregional therapy relative to systemic therapies, Jeyarajah et al. recently published the findings of a Delphi panel of experienced practitioners, including surgical oncologists, transplant surgeons, and hepatopancreatobiliary surgeons, concluding that SIRT with Y-90 microspheres may be effective at multiple points in the algorithm of liver-dominant mCRC management, including the complete treatment of small metastases, as first-line therapy for liver metastases either alone or in combination with chemotherapy, in combination with second- or third-line chemotherapy, and as salvage therapy for chemotherapy-refractory patients. The authors further recommended considering the various positions in therapy alongside the principle that SIRT should be introduced in the treatment algorithm to control liver tumor progression before the liver has been damaged severely by chemotherapy (Jeyarajah et al. 2020).

From a clinical perspective, the adverse event profiles of each agent, along with patient performance status, are likely to determine treatment choice (Argiles et al. 2019). While ECOG performance statuses 1–2 are considered negative prognostic factors for each intervention, the adverse event profile of SIRT using Y-90 resin microspheres is superior to both regorafenib and TAS-102, with a low incidence of all-grade or grade 3–4 AEs known to severely affect patient quality of life, such as diarrhea, hand–foot skin reaction, vomiting, or fatigue. The adverse event profile of SIRT is well established, and risk of complications from the procedure can be reduced with adequate treatment planning and patient selection (Sangro et al. 2017). SIRT may, therefore, be preferred in selected patients with colorectal liver metastases refractory to first- and second-line chemotherapy, especially when the AE profile of TAS-102 and regorafenib represents a disincentive for treatment with these agents. The incidence of the five potentially SIRT-related AEs recorded in the present study was low, with only grade 3+ REILD and grade 1–2 GI ulcer showing incidence rates above 5% in one study each.

Liver-related laboratory test abnormalities were not reported in a consistent manner across the included studies, and a meaningful analysis or presentation was not possible. A previous study of 606 patients with mCRC undergoing SIRT showed that a high proportion of patients had mild-to-moderate (mostly grade 1 or 2) baseline laboratory abnormalities prior to SIRT with Y-90 resin microspheres, including alkaline phosphatase, AST, albumin, and hemoglobin (Kennedy et al. 2015). While the study showed clinically significant increases in severe (grade 3 and 4) laboratory test values for total bilirubin, albumin, alkaline phosphatase, and aspartate aminotransferase after SIRT with Y-90 microspheres, all incidence rates were below 10% 90 days after treatment (Kennedy et al. 2015).

In indications such as mCRC in which patients are facing a poor prognosis with limited therapeutic options, the availability of locoregional options such as SIRT in addition to regorafenib and TAS-102 addresses a great unmet clinical need, especially in RAS mutant patients for whom anti-EGFR agents such as cetuximab or panitumumab are ineffective (Van Cutsem et al. 2015). Effective physician–patient communication is an essential element in ensuring appropriate treatment selection, and for setting expectations for treatment outcomes and adverse event incidence. As both regorafenib and TAS-102 are orally administered, patient adherence to the choice of treatment is also essential (Argiles et al. 2019). From the patient perspective, SIRT using Y-90 resin microspheres represents a relevant alternative with lower administration burden for the patient and no degradation of quality of life (Cosimelli et al. 2010). The adverse event profile of SIRT using Y-90 resin microspheres has also been established across the non-comparative evidence base in this indication for patients intolerant to chemotherapy and/or patients aged ≥ 70 years (Golfieri et al. 2015; Cosimelli et al. 2010; Kennedy et al. 2015; Saxena et al. 2015; Tohme et al. 2014; Lahti et al. 2015; Fendler et al. 2013; Maleux et al. 2016; Sofocleous et al. 2015; Nace et al. 2011).

## Conclusions

This systematic review and exploratory NMA of regorafenib, TAS-102, and SIRT using Y-90 resin microspheres in third-line mCRC demonstrate that all interventions are effective compared to BSC. Although SIRT was shown to have a greater OS benefit than systemic therapy for selected patients with liver-dominant colorectal metastases, this particular result needs to be interpreted with caution due to the differences in study design across interventions.

The improved adverse event profile of SIRT using Y-90 resin microspheres over systemic therapy should be considered in the physician and patient therapeutic decision-making process. Further research is needed to establish the relative costs of these interventions, and to determine the cost-effectiveness and budget impact of SIRT, regorafenib, and TAS-102 versus BSC.

## Electronic supplementary material

Below is the link to the electronic supplementary material.

Supplementary material 1 (DOCX 1036 kb)
