# Supplementary material for: Systematic review and network meta-analyses of third-line treatments for metastatic colorectal cancer
Source: J Cancer Res Clin Oncol. 2020 Jul 27;146(10):2575–87. doi: 10.1007/s00432-020-03315-6 (PMC7467965; doi:10.1007/s00432-020-03315-6)
Supplement: Supplementary file 1 — Supplementary material 1 (DOCX 1036 kb) [file 432_2020_3315_MOESM1_ESM.docx]

# Appendix

**Supplementary Table 1 Search terms for PubMed/EMBASE**


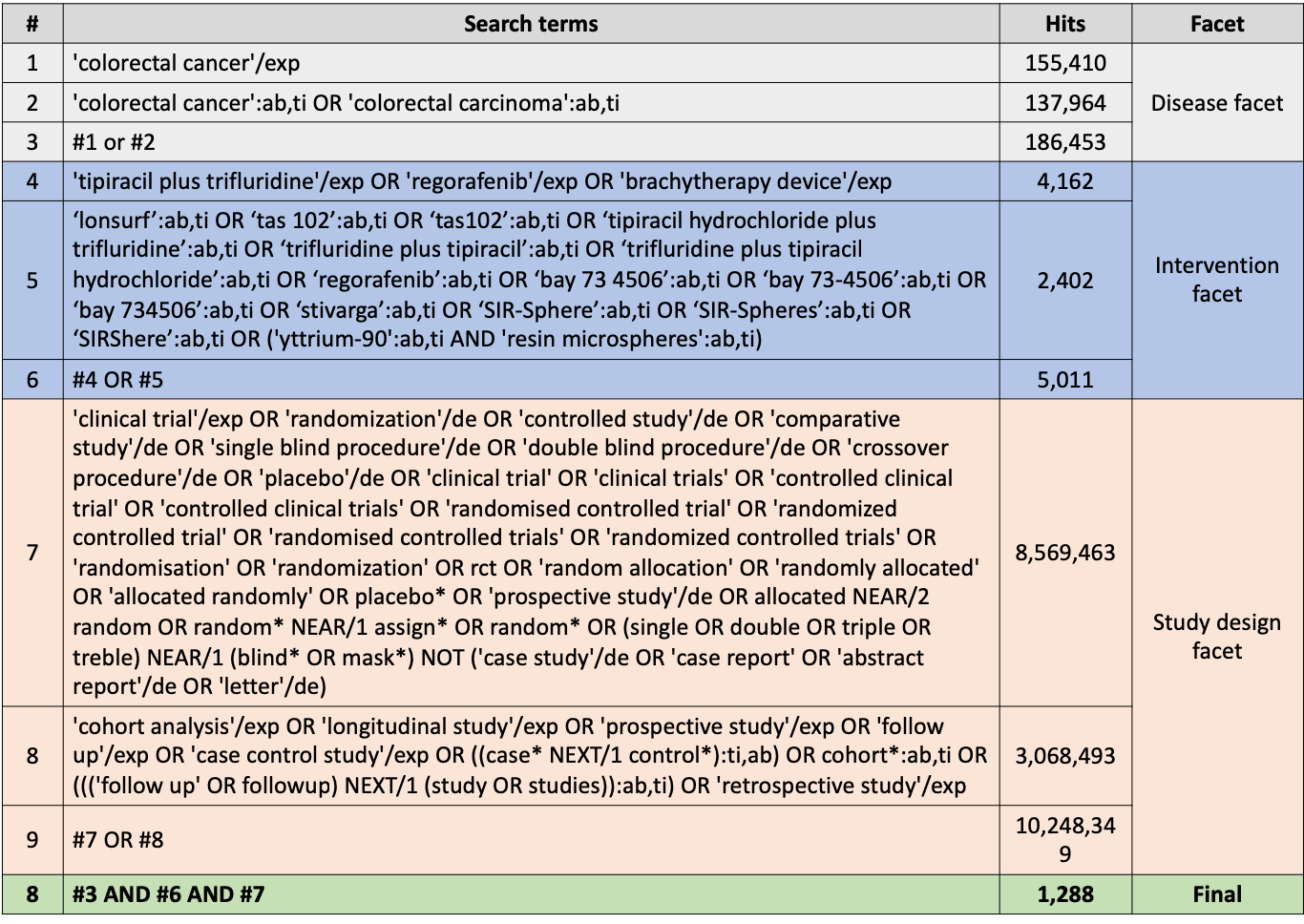


**Supplementary Table 2 Search terms for the Cochrane Library**


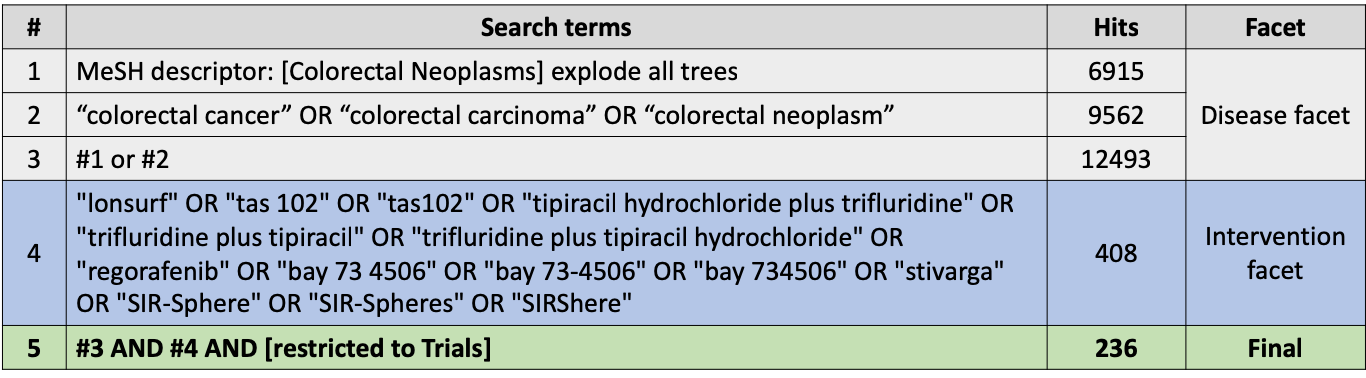


Supplementary Figure 1 Literature search flow diagram in line with the

Records identified through database searching
(n=1524)

Additional records identified through other sources
(n=0)

Records after duplicates removed
(n=1334)

Records screened
(n=1334)

Full-text articles assessed for eligibility
(n=40)

Studies included in synthesis
(n=7)

Records excluded
(n=1294)

Full-text articles excluded, with reasons
(n=33)

**Identification**

**Screening**

**Eligibility**

**Included**

Supplementary Table 3 Detailed eligibility criteria

| **Study** | **Eligibility criteria** |
| --- | --- |
| Bester 2012 | Patients had unresectable hepatic primary or metastatic cancer and liver-dominant tumour burden. Patients must demonstrate sufficient functional liver reserve and minimal extrahepatic disease (EHD), with a life expectancy of at least 3 months after the procedure. All patients were assessed using inclusion and exclusion criteria which form part of the pre-treatment assessment and workup:   - Acceptable patient history: - Unresectable primary or metastatic liver disease (tumour(s) not amenable to ablation or surgical resection) - Predicted life expectancy >=3 months - Liver-only or liver-dominant disease - Tumour burden <75% of liver volume - ECOG score =<2 - Child-Pugh class =<B - Sufficient hepatic reserve (bilirubin =<2.0 mg/dL, AST and ALT <5 times - upper limit of normal, albumin >3.5 mg/dL) - Adequate renal function (creatinine =<2.0 mg/dL) - Adequate blood count (granulocyte count =<1.5 x 10^9^/L, platelets =<75 x 10^9^/L) - Contraindications to 90Y radioembolisation:   - Limited hepatic reserve, irreversibly elevated bilirubin, extensive portal vein thrombosis or obstructed bile duct, ascites or clinical liver failure   - Excessive tumour burden with limited hepatic reserve   - Child-Pugh class C   - Previous extensive liver resection   - Concurrent or prior capecitabine chemotherapy (within 2 months prior to radioembolisation) |
| Grothey 2013 | Histological or cytological documentation of adenocarcinoma of the colon or rectum. They had to have received locally and currently approved standard therapies and to have disease progression during or within 3 months after the last administration of the last standard therapy or to have stopped standard therapy because of unacceptable toxic eff ects. Available standard therapies had to include as many of the following as were licensed: a fluoropyrimidine, oxaliplatin, irinotecan, and bevacizumab; and cetuximab or panitumumab for patients who had KRAS wild-type tumours. Patients had to be aged 18 years or older and have an ECOG ps of 0 or 1; life expectancy of at least 3 months; and adequate bone-marrow, liver, and renal function at the start of the trial. Patients could not participate if they had previously received regorafenib or had uncontrolled medical disorders. |
| Hendlisz 2010 | Patients with histologically proven adenocarcinoma of the colon or rectum metastasized to the liver only, not amenable to curative surgery or local ablation and resistant or intolerant to standard chemotherapy (FU, oxaliplatin, and irinotecan) |
| Li 2015 | Eligible patients had histologically or cytologically confirmed adenocarcinoma of the colon or rectum, with measurable or non-measurable metastatic disease according to RECIST. Patients had to have received at least two previous treatment lines, including a fluoropyrimidine plus oxaliplatin or irinotecan. Previous treatment with bevacizumab, cetuximab, or panitumumab was allowed but not mandatory. Patients had to have evidence of disease progression during or within 3 months after the last standard treatment (or within 6 months of stopping adjuvant oxaliplatin) or have stopped standard treatment because of unacceptable toxic effects. Patients had to be Asian adults (≥18 years of age) with an ECOG performance status of 0 or 1, a life expectancy of at least 3 months, and adequate bone marrow, liver, and renal function at the start of the trial. |
| Mayer 2015 | Patients with biopsy-documented adenocarcinoma of the colon or rectum were eligible if they had received at least two prior regimens of standard chemotherapies, which could have included adjuvant chemotherapy if a tumor had recurred within 6 months after the last administration of this therapy; if they had either tumor progression within 3 months after the last administration of chemotherapy; or if they had had clinically significant adverse events from standard chemotherapies that precluded the readministration of those therapies. Patients were also required to have received chemotherapy with each of the following agents: afluoropyrimidine, oxaliplatin, irinotecan, bevacizumab, and — for patients with KRAS wild-type tumors — cetuximab or panitumumab. In addition, patients had to be 18 years of age or older; have adequate bone-marrow, liver, and renal function; and have an ECOG PS of 0 or 1 |
| Seidensticker 2012 | All patients had liver-dominant mCRC and intrahepatic tumor progression as confirmed by imaging (computer tomography [CT]/magnetic resonance imaging [MRI]) as well as tumor markers (CEA) and/or clinical symptoms. |
| Yoshino 2012 | 20 years or older; previous treatment history of two or more regimens of standard chemotherapy; were refractory or intolerant to a fluoropyrimidine, irinotecan, and oxaliplatin; ECOG 0-2; |

**Supplementary Figure 2 Trace and marginal density plots of the log hazard ratios for best supportive care, regorafenib, SIRT, and TAS-102 versus best supportive care in the random effects network meta analysis**


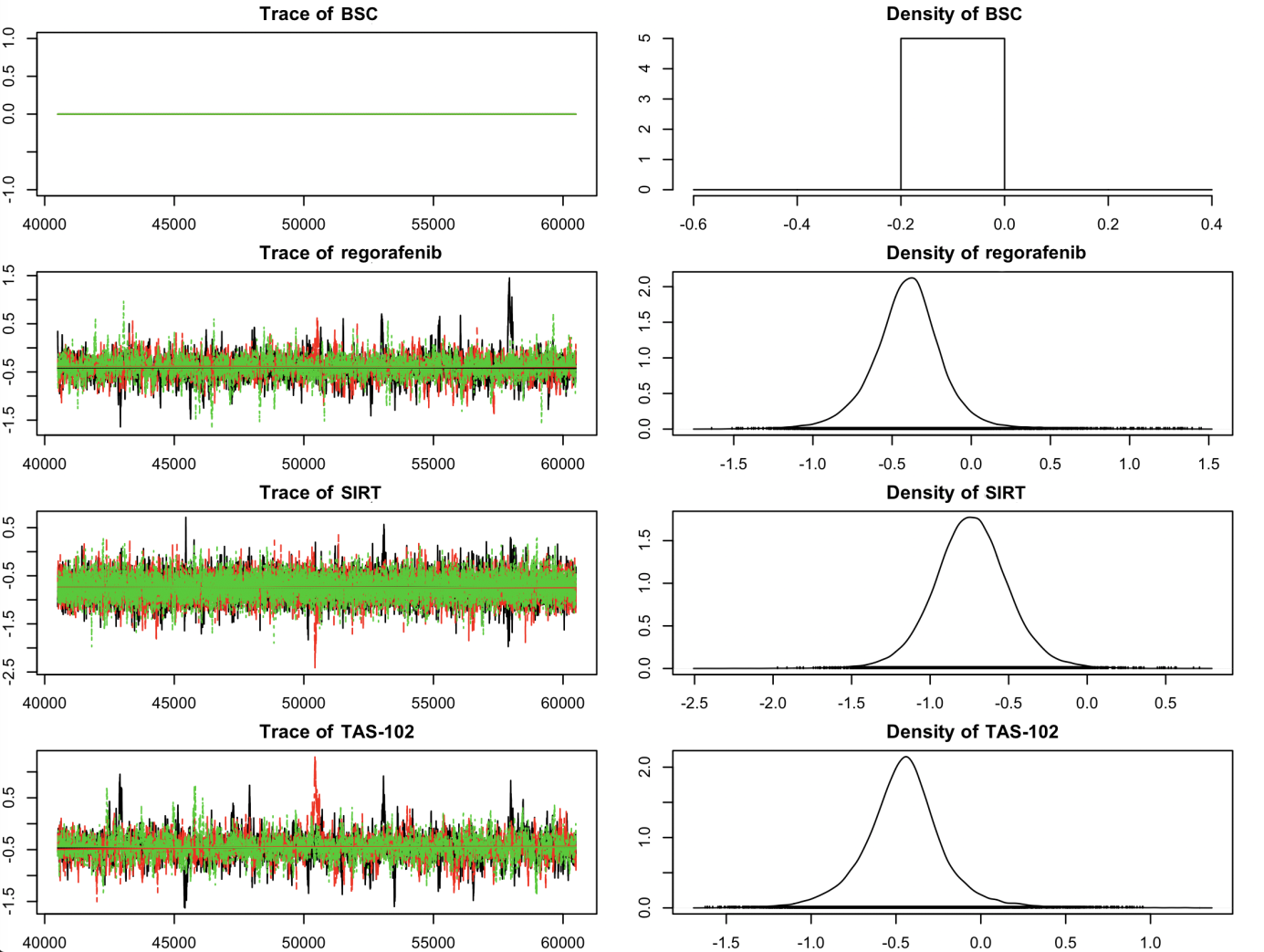


**Supplementary Figure 3 Gelman-Rubin plots showing chain convergence with increasing model iterations after 20,000 burn-in iterations**


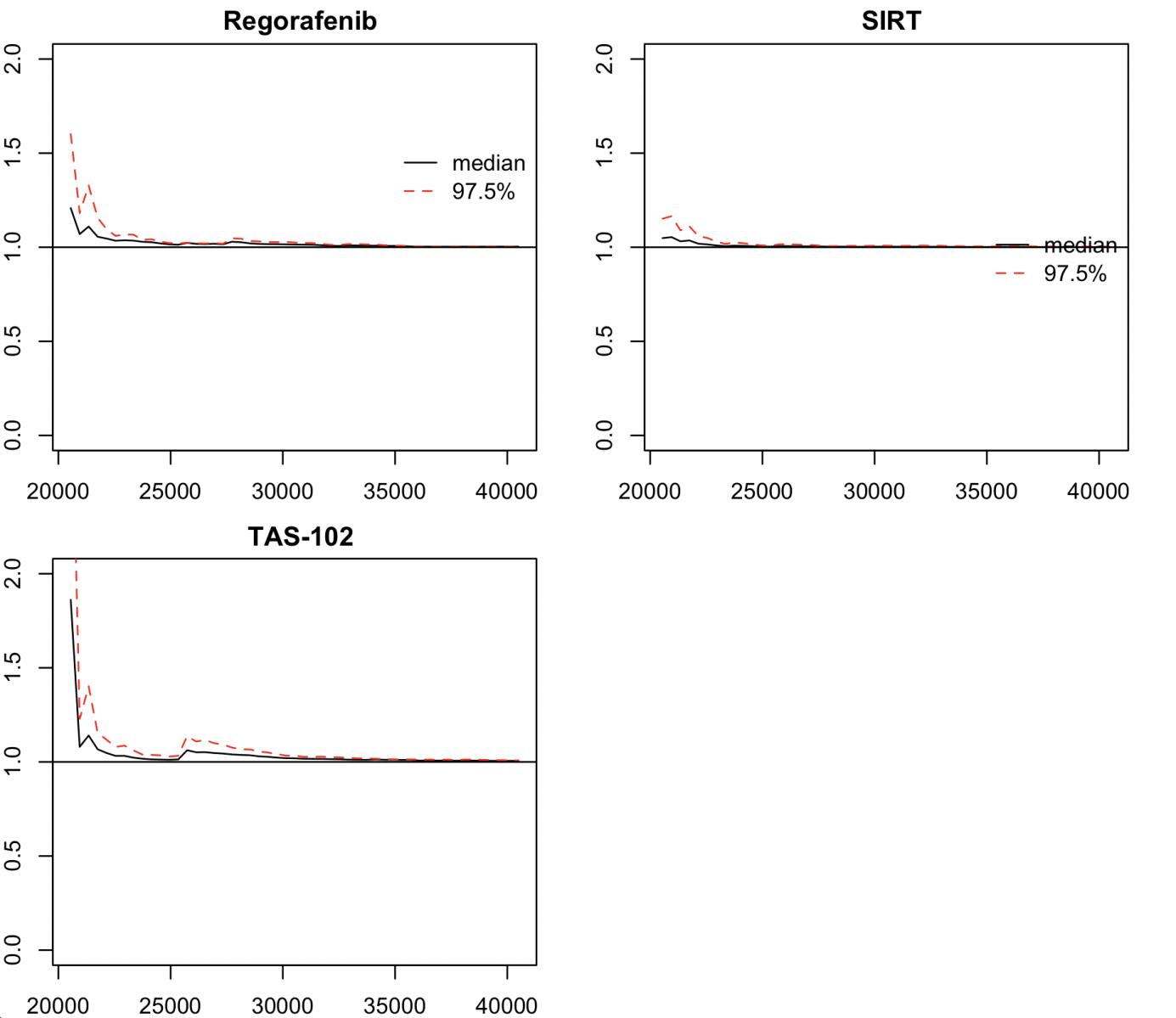


Supplementary Figure 4 Overall survival hazard ratios for SIRT relative to best supportive care from sensitivity analyses in which non-randomized study variance was inflated by a factor of 1/w_j_ with w_j_ set to a range of values between 0 and 1


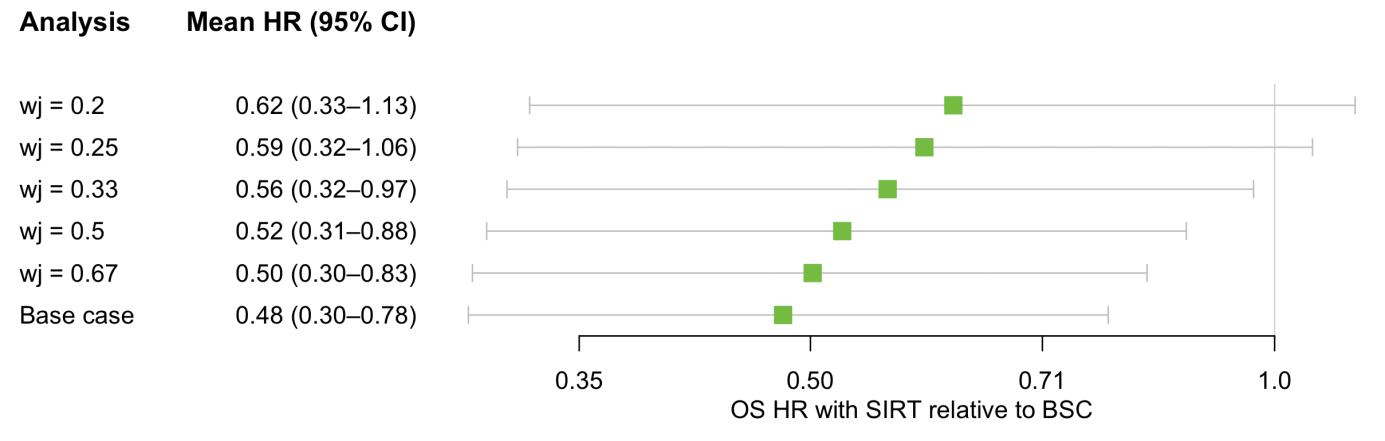


**Abbreviations:** BSC, best supportive care; HR, hazard ratio; OS, overall survival; SIRT, selective internal radiation therapy.
